# Assessing the Malignancy Risk of a Meningioma by Its Location

**DOI:** 10.7759/cureus.31213

**Published:** 2022-11-07

**Authors:** Luis A Rodríguez-Hernández, Rodrigo Uribe-Pacheco, Michel Mondragon-Soto, Juan Alvaro-Heredia, Ignacio Reyes-Moreno, Humberto Montano-Tello, Guillermo A Gutierrez-Aceves, Vicente Guerrero-Juarez, Elvira Castro-Martinez, Alberto Gonzalez-Aguilar

**Affiliations:** 1 Neurosurgery Department, Instituto Nacional de Neurología y Neurocirugía "Manuel Velasco Suárez", Mexico City, MEX; 2 Neurosurgery Department, Instituto Nacional de Neurología y Neurocirugía "Manuel Velasco Suárez", Mexico, MEX; 3 Neurosurgery, Neurology and Neurosurgery National Institute, Mexico City, MEX; 4 Neuroradiosurgery, Neurology and Neurosurgery National Institute, Mexico City, MEX; 5 Neurology, Neurology and Neurosurgery National Institute, Mexico City, MEX

**Keywords:** benign tumors, tumor, clinical study, malignancy, meningioma

## Abstract

Background: Meningiomas represent 30% of primary intracranial tumors. The current incidence is up to 4.5 cases per 100,000 habitants worldwide. Although there is no prognostic difference among benign histopathological subtypes, atypical meningiomas and malignant meningiomas (WHO grade II and III respectively) may extend to the adjacent brain parenchyma, dura mater, and osseous tissue with a recurrence score (21-49%). This manuscript analyzes the malignancy risk according to neoplastic localization through a logistic retrospective analysis from a total sample of 452 patients with grade I, II, and III (WHO) meningiomas.

Methods: Detailed data collection through a three-year retrospective analysis (January 2008 to December 2011) was applied at Mexico’s National Neurology and Neurosurgery Institute including patients with intracranial or spinal-cord meningioma, preoperative imaging study availability and post-surgical histopathological diagnosis. Formal written consent was not required with a waiver by the appropriate national research ethics committee in accordance with the provisions of the regulations of the general health law of Mexico.

Results: Convexity lesions displayed an increased risk of malignancy turning for non-benign meningiomas with an odds ratio of 3.1 (95% CI 1.6 to 5.7, p=0.0002) meanwhile skull-base meningiomas present an inverse risk with an odds ratio of 0.4 (95% CI 0.2 to 0.9, p=0.02), as well as spinal-cord meningiomas with an odds ratio of 0.3 (95% CI 0.1 to 0.9).

Conclusion: Skull base and spinal cord meningiomas usually have benign behavior, meanwhile grade II or III meningiomas within this location are rare. The present work provides an additional criterion for decision making, according to the meningioma’s location.

## Introduction

Meningiomas achieve about 30% of primary brain tumors overall, with an adjusted annual incidence of 4.5 cases per 100,000 people [1]. These tumors are more common in the elder population with a peak incidence in the seventh decade of life. Benign meningiomas (Grade I) are classified by the World Health Organization (WHO) according to their histopathological traits: meningothelial, fibroblastic, transitional, psammomatous, angiomatous, secretory, microcystic, lymphoplasmacyte-rich and metaplastic [2]. Although there is no prognostic difference between benign subtypes, atypical (Grade II) and malignant (Grade III) tumors, they all can invade adjacent brain parenchyma, dural sinuses, dura mater, and bone [3]. Appropriation of brain parenchyma is a significant recurrence factor with 21-49% recurrence rate, compared to benign meningiomas (with a recurrence probability of 7-20%) [4-6]. Atypical meningiomas have higher mortality and morbidity compared to their benign counterpart [7-9]. Notwithstanding, one of the most crucial objectives for physicians is to identify patients who have an atypical meningioma or anaplastic meningioma due to the risks this histopathological diagnosis conveys in prognosis. Previous studies have addressed this paradigm seeking to differentiate benign and non-benign lesions, particularly with the Diffusing-wave spectroscopy (DWS) sequence on brain magnetic resonance imaging [10] brain single photon-emission computed tomography (SPECT), and brain positron emission tomography (PET) with no concluding results. The purpose of this study is to predict the malignancy relative risk for atypical/malignant meningiomas based primarily on its onset location.

## Materials and methods

Detailed data collection took place through a three-year retrospective analysis (January 2008 to December 2011), where inclusion criteria for intracranial and spinal-cord meningioma were applied at Mexico’s National neurology and neurosurgery institute, well known as a tertiary-care concentration hospital sponsored by Mexico´s government department of health. Due to its volume of patients, scientific stand, human resources, and infrastructure, it is considered as the cutting-edge national institution of neurological diseases in our country.

The inclusion criteria considered: patients over 15 years old with intracranial or spinal-cord meningioma, with a preoperative imaging study available for evaluation (computed tomography and/or magnetic resonance imaging), and post-surgical resection histopathological diagnosis. The exclusion criteria involved: patients without preoperative images, and incomplete sociodemographic information. Sociodemographic data were recorded for all patients (age, sex, location, sub-histology, and degree of malignancy). Formal written informed consent was not required with a waiver by the appropriate institutional review board (IRB) and/or national research ethics committee in accordance with the provisions of the Regulations of the General Health Law of Mexico in the field of Health Research in its article 17, the present research study is considered to be of less than minimal risk, since only information was taken from the electronic file, in addition to the fact that data that could allow the identification of any of the patients is never disclosed.

Patients were classified according to 2007 WHO classification of brain tumors into two groups: Benign meningiomas (Grade I) and non-benign meningiomas (grade II and III meningiomas). Meningiomas were as well classified into six different groups according to their location: convexity, parasagittal, skull-base, posterior fossa, intraventricular, and spinal cord, respectively. A logistic regression analysis was performed with odds ratio calculation to estimate relative risk and its correlation with the meningioma’s location.

## Results

A total of 452 patients diagnosed with meningiomas were included during a 47-month period, out of which 231 patients met all the inclusion criteria for this study. The mean age on diagnosis was 49.3 years (95% CI 47.5 to 51.2). One hundred and fifty-seven (67.9%) were female, 74 (32.1%) were male, and the female/male ratio was 2.1:1.

According to the grade of malignancy, 74.4% were meningiomas Grade I (172 patients), and 25.5% were non-benign meningiomas (59 patients), either Grade II or III (WHO). The most frequent lesion distribution was identified in the convexity in 78 patients (33.8%), meanwhile, the rest were located parasagittal in 36 (15.6%), skull-base in 63 (27.3%), posterior fossa in 29 (12.6%), spinal-cord in 21 (9.1%), and intraventricular in four patients (1.7%). Spearman's correlation analysis for age/grade of malignancy and sex/grade of malignancy showed a non-statistically significant correlation (r2 = -0.0221, 95% CI -0.1 to 0.1 and r2 = -0.08, 95% CI 0.2 to 0.04, respectively). Logistic regression analysis evaluating the relative risk of benign and non-benign meningiomas according to the initial location (Tables 1, 2).

**Table 1:** Odds ratio of malignancy by age, sex, and location. OR: Odds Ratio; CI: Confidence Interval

Age/Sex	OR	CI 95%	p Value
Age <50 years	0.7	0.4 - 1.4	0.385
Male	1.2	0.6 - 1.2	0.161
Female	1		
Convexity	3.2	1.8 - 6.0	<0.001
Parasagittal	1.1	0.5 - 2.4	0.823
Posterior Fossa	0.4	0.1 - 1.3	0.1
Skull base	0.4	0.2 - 0.9	0.021
Intraventricular	0.9	0.1 - 9.5	0.98
Spinal Cord	0.3	0.1 - 0.9	0.048

**Table 2:** Relationship between location and degree of malignancy

Location	Meningioma grade (WHO)		Total
	I	II/III	n (%)
	n (%)	n (%)	
Convexity	46(27.2)	32(55.2)	78(34.4)
Parasagital	26(15.4)	10(17.2)	36(15.9)
Skull Base	53(31.4)	10(17.2)	63(27.8)
Posterior Fossa	25(14.8)	4(6.9)	29(12.8)
Intraventricular	3(1.8)	1(1.7)	4(1.8)
Spinal cord	19(11.2)	2(3.4)	21(9.3)
Total	169(100)	58(100)	227(100)

Convexity-located meningiomas displayed an increased risk of non-benign meningiomas with an odds ratio (OR) of 3.1 (95% CI 1.6 to 5.7, p=0.0002), meanwhile, skull-base meningiomas had an inverse risk of non-benign meningiomas with an OR 0.4 (95% CI 0.2 to 0.9, p=0.02) as well as spinal-cord meningiomas with an OR 0.3 (95% CI 0.1 to 0.9). The remainder locations showed no statistically significant differences.

## Discussion

According to our results, patients with intracranial or spinal-cord meningiomas present a clinical onset at an earlier age (49.3 years old) contrary to the reported in the literature (mean age of clinical onset of 63 years old) [11]. One of the largest case series reported is the French Brain Tumor Group with 43,929 cases, where 32.3% corresponded to meningiomas, and the age of presentation was 57 years [12]. In Mexico there is no accurate statistic annual report of the population’s frequency of meningiomas, González-López reported this brain tumor as the third most-frequent tumor after gliomas and adenomas. Meningiomas accounted for 22% of all primary brain tumors [13].

A possible explanation for this epidemiological phenomenon could be a different molecular biological expression that our population may present; however, this assumption is difficult to prove at the moment, since there are no genetic studies of our population up to date. The frequency of meningiomas in accordance with the WHO 2007 brain tumor classification, 92% are grade I and the remainder 8% are Grade II and III [14-15]. In clinical practice, it is relevant to identify atypical and anaplastic meningiomas, due to their unfavorable prognosis despite a gross-total resection. These malignant meningiomas need adjuvant therapy with radiotherapy to avoid or delay their expected recurrence. Our studied population showed a frequency of non-benign meningiomas of 25.5% and 74.5% benign meningiomas with average aging in adulthood of 49.3 years old, yet we have a number of adolescent cases in which a higher frequency of non-benign meningiomas was found [16]. The WHO has released two evaluations on histopathology standards (the first one in 2000 and the most recent in 2007). Accordingly, reported series published after 2007 show that up to 30% of meningiomas can be non-benign. It is possible that the previous series underdiagnosed grade II meningiomas, considering that both biology and histopathological specifications were considered to update its definition for diagnosis [17].

A vast number of meningioma classifications with different purposes exist, that often are neither relevant nor well-known for non-neurosurgery physicians such as primary-care and emergency-care specialists. Considering this, we used a simplified classification to standardize meningiomas that not only may be easy to use but possibly correlate with recurrence risk. We found that the most frequent site of meningiomas within this sample of patient was: convexity (33.8%), parasagittal (15.6%), skull base (27.3%), posterior fossa (12.6%), spinal cord (9.1%) and intraventricular (1.7%); these results are similar to those reported in the literature (2,16,17). The main objective of this study was to identify the risk of recurrence, expressed through the odds ratio of non-benign meningiomas in relation to their location of onset through a logistic regression analysis. Convexity meningiomas showed an OR of 3.1 (95% CI 1.6-5.7, p = 0.0002) of presenting non-benign meningiomas (atypical or anaplastic), while meningiomas of the skull-base and spinal-cord had an OR 0.4 (95% CI to 0.2 to 0.9 and p = 0.02) and OR 0.3 respectively (95% CI to 0.1 to 0.9 and p = 0.04), indicating low-probability of non-benign meningiomas in these two locations.

On the other hand, the natural history of meningiomas has been extensively studied, Nakasu et al. described meningiomas may have three types of growth behavior over time: meningiomas with exponential growth (grows progressively in a constant fraction), linear growth (gradual growth rate with a decrease in the rate of cell proliferation and/or increase in the rate of cell death), and without growth [18]. Meningiomas are more frequent in convexity and parasagittal locations, both in which their growth behavior may be influenced by the fact of bone structures on one plane only, and are not a limiting factor for expansion or growth. Also, cortical gyri, sulcus, and parenchyma as well as vascular structures are much more malleable to compression, which may explain why this tumor location is later diagnosed than skull-base and/or spinal cord, primarily because the symptoms in these last two tend to manifest earlier since bone structures surround more than one anatomic plane and both structural compression and parenchymal or nerve compromise takes place earlier as well [18-19].

Another possible explanation for the progressive growth-tendency and higher malignancy in both convexity and parasagittal meningiomas is the adaptive ability of tumoral tissue to continuously decrease oxygenation over time, explained by the relative compression unrelated to the diploe plane. This over-the-time decrease of oxygen supply favors of non-acute, non-ischemic, yet hypoxic environment which may convey a potential risk of anaplastic transformation by the sum of new mutations related to chronic cellular hypoxia [18-19] and angiogenesis [20]. In meningiomas, the neoangiogenic trait has been reported as a marker of malignancy [21]. Several studies report in meningiomas, that the expression of hypoxia-induced factor (HIF) favors the expression of vascular endothelial growth factor (VEGF), which is a growth factor that has a correlation directly proportional to the degree of malignancy of tumors and seems to be part of the molecular steps for both neoangiogenesis and anaplasia [22].

Remarkably, skull base and spinal-cord meningiomas’ growth is limited by shorter space and bone structures. Oxygen supply may not be compromised over a time period that may provide hypoxia adaption, which could explain their lower risk of non-benign meningiomas. A study led by Hashimoto et al. describes that tumors of the skull base have slower growth in comparison to other intracranial locations of meningiomas [23]. In this series the proliferation rate was measured by immunohistochemical examination with MIB-1 antibody, used as a marker for tumor proliferative potential, where MIB-1 of 2.7% was found for intracranial meningiomas located in other than the skull base, meanwhile, skull-base meningiomas rate was 2% (p 0.0013), these differences are statistically significant [22-23]. Another deduction that could be drawn from this is that tumors have different molecular profiles. Al-Rashed et al. reported that skull-base meningiomas have a lower frequency of chromosome-1 loss, which is considered to be associated with a higher growth rate [23].

To our knowledge, there is no previous study that includes spinal cord meningiomas and correlates the location with recurrence risk along with intracranial meningiomas. There are no previous studies that approach their growth behavior, but we assume that the same principle of skull-base meningiomas may take place. Being confined to the spinal canal may limit the expansion of tumors favoring that the size and blood flow are constant without compromising gene stability. Our manuscript is the second report in the scientific literature that associates tumoral location related to malignancy. Nevertheless, this is the first report that includes spinal-cord meningiomas as well as intracranial within Mexico’s population. 

In this light, the primary limitations of the present manuscript are that it is a retrospective study and neither molecular nor genetic analysis, except by histological correlation, is considered with risk of malignancy or recurrence. Also, the resection extent was not considered within the variables for malignancy or turning from a grade I to II or III. A prospective cohort including volumetric, genetic, and molecular analysis should be employed in order to correlate specific parameters that may influence the development of anaplasia in meningiomas from a molecular basis.

## Conclusions

The cornerstone of this document is to conclude the probability of non-benign meningiomas turning malignant as a higher risk score in the convexity and inversely, with a low probability of non-benign meningiomas at the skull base and spinal cord. The malignancy risk related to neoplastic localization is used as an additional variable to be considered in the decision-making of surveillance and complementary meningioma approach.
